# Incidence of herpes zoster and postherpetic neuralgia in Italian adults aged ≥50 years: A prospective study^☆^

**DOI:** 10.1016/j.pmedr.2019.100882

**Published:** 2019-04-24

**Authors:** Andrea Salvetti, Vincenzo Ferrari, Remigio Garofalo, Pietro Gazzaniga, Alessandro Guerroni, Antonio Metrucci, Aurelio Sessa, Maria Loretta Severi, Giulio Nati, Mauro Ruggeri, Alessandro Rossi, Laura Cappellari, Kusuma Gopala, Roberta Tosatto, Benedetto Simone

**Affiliations:** a Investigator site, Piazza Ponchielli 1, Toscana, Grosseto, GR 58100, Italy; b Investigator site, Via Terranova 2, Puglia, Parabita, LE 73052, Italy; c Investigator site, Via B. di Fredi 24, Toscana, Civitella Paganico, GR 58045, Italy; d Investigator site, Via Garibaldi 1, Piemonte, Frugarolo, AL 15065, Italy; e Investigator site, Via Vittorio Veneto 44/A, Lombardia, Sesto Calende, VA 21018, Italy; f Investigator site, Viale della Repubblica snc, Puglia, Cutrofiano, LE 73020, Italy; g Investigator site, Via Cavour 26, Lombardia, Arcisate, VA, 21051, Italy; h Investigator site, Via Cechov 3/Q, Lazio, Roma, RM 00142, Italy; i Investigator site, Via Pietro Tacchini 7, Lazio, Roma, RM 00197, Italy; j Investigator site, Central Site, Società Italiana di Medicina Generale (SIMG), Via del Pignoncino 9 SIMG, Toscana, Firenze, FI 50142, Italy; k GSK, Via Fleming 2, Veneto, Verona, VR 37135, Italy; l GSK, Embassy Links, Cunningham Road, SRT Road, #5, Bangalore 560052, India; m GSK, Stockley park, Iron Bridge Rd N, UB11 1BT, United Kingdom

**Keywords:** Burden of disease, Herpes zoster, Postherpetic neuralgia, Primary care setting, Prospective study, Italy

## Abstract

Herpes zoster (HZ) mainly affects older adults and immunocompromised individuals and is usually characterized by a unilateral painful skin rash. Its most common complication, postherpetic neuralgia (PHN), may cause chronic debilitating pain lasting for months or years. This study (ClinicalTrials.gov Identifier: NCT01772160) aimed to estimate the HZ incidence and the proportion of HZ patients with PHN in the Italian population aged 50 years or older.

From 2013 to 2016, HZ-patients were recruited when presenting with acute HZ at 75 reporting general practitioners in Italy, covering 43,875 persons aged ≥50 years. PHN was defined as ‘worst pain’ rated ≥ 3 on the Zoster Brief Pain Inventory persisting or appearing over 90 days after rash onset.

The overall HZ incidence rate per 1000 person-years (PY) was estimated as 6.46 (95% confidence interval [CI]: 5.99–6.95), increasing with age to 9.12/1000 PY (95% CI: 7.50–10.99) in 75–79 year-olds. Among 391 HZ-patients who completed the study, the overall proportion with PHN was 10.23% (95% CI: 7.41–13.67) and the highest proportion was 15.56% (95% CI: 6.49–29.46) for the 75–79 year-olds. Among the 128 patients (32.7%) who reported at least one pre-existing medical condition, 35.9% reported diabetes mellitus and 32.0% reported emotional problems, stress or depression.

The study confirms previous findings that HZ and PHN cause an important clinical burden in older Italian adults. It also confirmed the age-related increasing risk of HZ and PHN.

## Highlights

• HZ has a strong incidence in the Italian population, the 2nd oldest worldwide.
• The estimated overall HZ incidence rate was 6.46 per 1000 person-years.
• The most common complication, PHN, was encountered by 10.23% of HZ patients.
• Strengths of the study are its prospective design and its large sample.
• Limitation: incidence may be underestimated due to cases missed in primary care.

## 1 Introduction

Varicella-zoster virus (VZV) causes varicella infection with most cases occurring in young children. After resolution of the varicella infection, VZV remains lifelong latent in cranial nerve or dorsal root ganglia and may reactivate later in life causing herpes zoster (HZ) infection. The typical manifestation of HZ is a unilateral, dermatomal vesicular rash, which is painful and/or pruritic. Often, the onset of rash is preceded by symptoms up to several days in advance, especially the so-called prodromal pain (Cohen, 2013; Gershon and Gershon, 2013).

The individual lifetime risk of HZ is often stated to be about 30% (Pinchinat et al., 2013). It is well established that VZV-specific cell-mediated immunity (CMI), and not anti-VZV antibodies, is essential for maintaining latency of VZV (Gershon et al., 2010). It is also clear that reactivation is associated with a decline in CMI, which commonly occurs naturally with aging or may be a consequence of immunosuppression due to disease or treatment (Gershon et al., 2010). Consistent with this, aging is the most common risk factor for HZ, in particular from the age of 50, concurrently with usual acceleration of natural immunosenescence (Cohen, 2013).

Most cases of HZ resolve within four weeks, but about 25% of HZ patients experience various complications which may retard full recovery. Acute complications include various neurologic conditions, disseminated skin disease and several ophthalmologic problems (Cohen, 2013). The most common complication is postherpetic neuralgia (PHN), i.e., chronic pain which may last months or years. The pain may be severe and have deleterious impacts on the patients' sleep, activities of daily living and overall quality of life (Cohen, 2013; Gershon and Gershon, 2013; Johnson, 2010).

The primary treatment of HZ is antiviral therapy. If such therapy is initiated within 72 h of rash onset, it may advance the resolution of rash lesions and reduce the formation of new, lessen viral shedding and diminish the severity of acute pain. Analgesics, including opioids, can also be used for pain management. However, there is no evidence that antivirals have any effect on the development of PHN (Cohen, 2013; Gershon and Gershon, 2013).

Prevention of HZ has been possible since 2006, when a live attenuated vaccine was first licensed in the US (Ansaldi et al., 2016; US-FDA, 2006). In 2017, an alternative, recombinant, adjuvanted zoster vaccine (RZV) was licensed in the US and in 2018 in the European Union (EU) (EMA, 2018; US-FDA, 2017). RZV is currently being assessed for licensure in many other countries and regions.

A recent systematic literature review found similar HZ incidence rates across all countries in the European Economic Area (the EU plus Iceland, Norway and Switzerland) for which studies were available. For all ages combined, a mean incidence of 3.4 per 1000 person-years (PY) was found. The studies consistently found that the incidence increased with age and rather steeply so after age 50. For individuals aged 20–50, the mean rate was 2.5/1000 PY increasing to 7–8/1000 PY for individuals aged ≥50 years and up to 10/1000 PY for those aged ≥80 (Pinchinat et al., 2013).

Among HZ patients, PHN and other HZ-related complications also occur more frequently with advancing age (Johnson, 2010; McElhaney, 2010; Yawn et al., 2007). As the number and proportion of older people grows all over Europe, the public health burden of HZ will likely increase in the coming years (Pinchinat et al., 2013; United Nations Department of Economic and Social Affairs, 2017; Varghese et al., 2017).

Italy has the second highest proportion of older adults worldwide. At the study start, the data on the incidence and burden of HZ in Italy were limited to two retrospective database studies (Gabutti et al., 2010; Gialloreti et al., 2010) and to a small prospective study with only 46 HZ patients (Di Legami et al., 2007). The primary objective of the present study was to estimate prospectively the incidence of a first episode of HZ in Italians aged 50 years or above in the primary care setting. Secondary objectives were to estimate the proportion of HZ patients developing PHN and assessing possible risk factors for PHN.

## 2 Material and methods

This was a prospective observational study carried out in the primary care setting (ClinicalTrials.gov Identifier: NCT01772160). The calculation of sample size for the descriptive statistical analyses was based on considerations about the acceptable degree of precision of the estimated HZ incidence. With an anticipated incidence rate of 6–7/1000 PY, a 95% confidence interval (CI) of width 1.0/1000 PY was deemed to be a satisfactory precision. To achieve this, around 700 HZ cases would be required, occurring over approximately 100,000 PY.

**Fig. 1:**
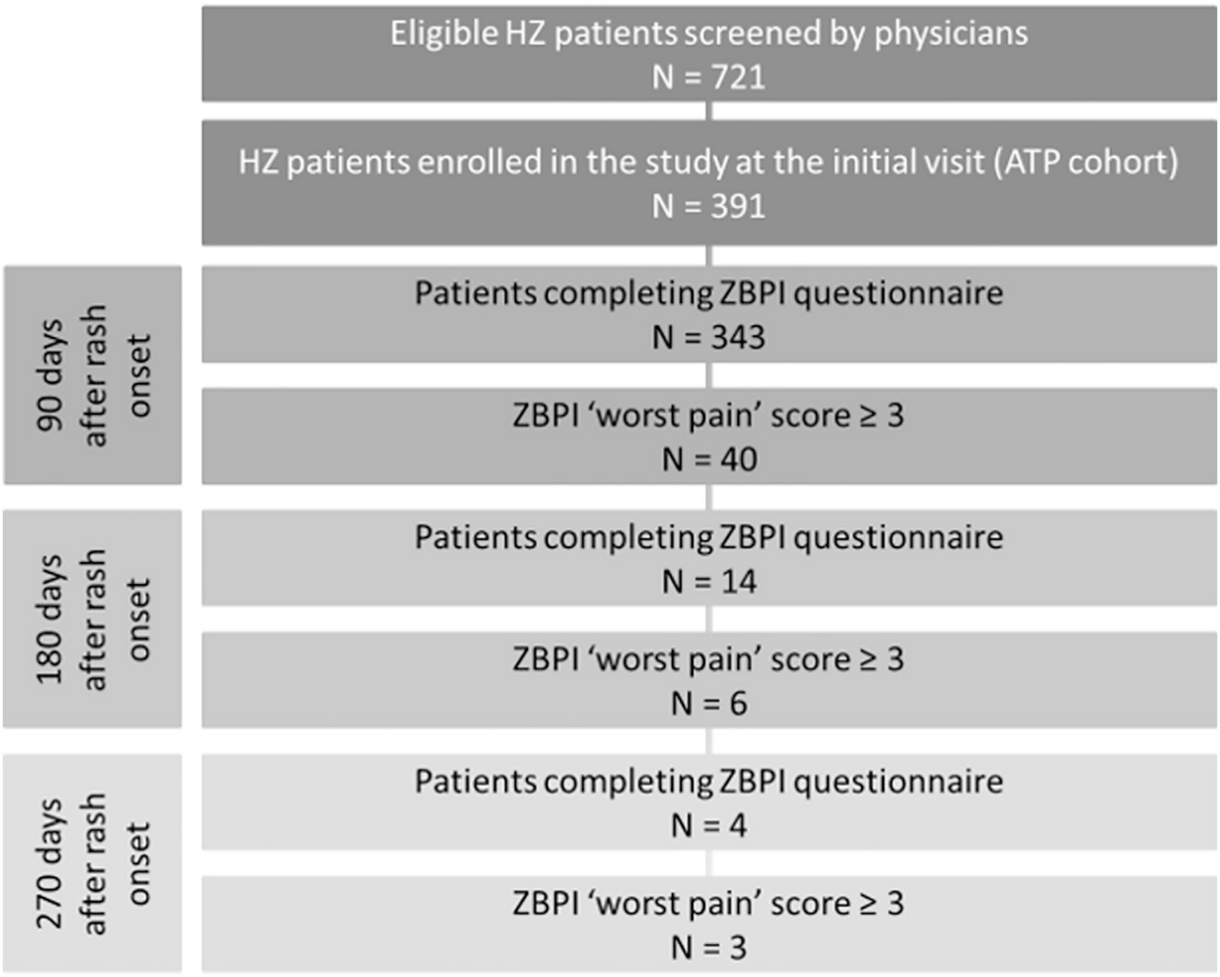
Flow chart. HZ = Herpes zoster; ZBPI = zoster brief pain inventory.

The participating physicians were general practitioners (GPs) belonging to 9 networks defined as a group of GPs located in the same town or province and linked to the same ethics committee. The selection of networks and the related GPs was done by the Società Italiana di Medicina Generale (SIMG). Approximately 75 GPs agreed to participate in the study, each with an average pool of 1300 registered individuals aged ≥15 years. Around 45% of the individuals aged ≥15 years registered with a GP practice are 50 years old or older, so the total number of individuals aged ≥50 years surveyed for the incidence of HZ would be approximately 43,875 and a hypothetical study period of about 2 years and 4 months would be required to cover 100,000 PY. In practice, it could not be expected that all the screened HZ patients would accept to be enrolled in the study. Therefore, the study period was pre-determined to last from the date of study start until the date when a target enrollment of approximately 400 HZ patients aged ≥50 years had been reached followed by the period of follow-up required to assess the secondary objectives of the study, i.e., estimating the proportion of patients with PHN 90, 180 and 270 days, respectively, after the onset of HZ (Fig. 1).

A case of HZ was defined as new unilateral pain (including also allodynia and pruritus) accompanied by unilateral rash and no alternative diagnosis. The severity of pain was assessed by a pain intensity index, more specifically question 3 (‘worst pain’) of the zoster brief pain inventory (ZBPI) (Coplan et al., 2004), a method which has been extensively used in previous studies e.g. (Drolet et al., 2010; Gater et al., 2014; Schmader et al., 2007; Schmidt-Ott et al., 2018; Yanni et al., 2018). The 11-point scale (0 to 10) was subdivided into 3 categories: no or mild pain (0–2); moderate pain (3–6); severe pain (7–10). Clinically relevant PHN was defined as the presence of HZ-associated moderate or severe ‘worst pain’ persisting or appearing more than 90 days after onset of the rash, i.e., a ‘worst pain’ score ≥ 3 on the ZBPI index.

### 2.1 Estimation of the HZ incidence

For the calculation of the HZ incidence (number of HZ episodes per 1000 PY), the total population (denominator) was defined as the number of individuals aged ≥50 years registered with the participating GPs. An active GP center was a participating center, which, during the study provided monthly logbooks recording the number of individuals aged ≥50 with an acute episode of HZ referring to the GP center that month, regardless of the occurrence or not of an episode of HZ. Active GPs who interrupted their participation before the end of the study provided monthly logbooks until the last month of their participation.

With *duration* calculated as the number of days for which the logbook data on the screened number of HZ cases were available for each center, the HZ incidence was estimated using the following formula:

$$\mathrm{Incidence of}\;\mathrm{HZ}=\frac{\mathrm{Number of}\;\mathrm{HZ}\;\mathrm{ca}s\mathrm{es}\;\mathrm{screened}\;\left(721\right)\;\mathrm{as}\;\mathrm{per}\;\mathrm{the logbook}\;\left(\geq 50\;\mathrm{years}\right)}{\mathrm{sum}\;\mathrm{of}\;\left(\mathrm{Total population}\;\left(\geq 50\;\mathrm{years}\right)\times \mathrm{duration}\left(\mathrm{days}\right)\right)\;\mathrm{across}\;\mathrm{all}\;\mathrm{centres}}\times 365.25\times 1000$$

The incidence of HZ was estimated overall, by gender and by age groups (50–54, 55–59, 60–64, 65–69, 70–74, 75–79, and ≥80 years).

### 2.2 Inclusion in the study cohort

Patients aged ≥50 years and diagnosed with a first episode of acute HZ were eligible for inclusion in the study cohort, either if the diagnosis was made during the initial consultation or if the visit was a secondary consultation for the HZ and the diagnosis had been made less than 7 days before in another setting (e.g., an emergency room or a specialist dermatological practice). Eligibility also depended on the GP's assessment of the patient's ability to comply with the study procedures (in particular completing self-administered questionnaires) and on the patient giving written informed consent to be enrolled. The only exclusion criterion was concurrent participation in an interventional clinical study. For screened patients not enrolled in the study, no further data were collected.

For patients enrolled in the study, the GP completed a case report form with demographic data, medical history, HZ vaccination status and clinical HZ information. The GP was also available for assistance if needed by the patient in completing the ZBPI and other questionnaires during the initial visit. Enrolled patients were asked to complete the ZBPI at Days 15, 30, 60 and 90 after the initial visit. If a patient on Day 90 rated the ‘worst pain’ item of the ZBPI as ≤2, follow-up was terminated. Patients with PHN at Day 90 were asked to continue completing the ZBPI on Days 120, 150 and 180 and if their ‘worst pain’ assessment on the ZBPI questionnaire at Day 180 was ≤2, the follow-up was terminated. For patients with PHN at Day 180 follow-up was continued and they were asked to complete ZBPI assessments at Days 210, 240 and 270. No patient was followed after Day 270.

### 2.3 Proportion of HZ patients with PHN

The proportions of all enrolled HZ patients with PHN at Days 90, 180 and 270 with exact 95% CIs were calculated. These proportions were calculated overall and stratified by gender, age group and severity of HZ at the initial visit. The proportion with PHN at Day 90 was also calculated among the patients that completed the ZBPI at this time point.

### 2.4 Exploratory analyses of risk factors for PHN

To describe the association between development of PHN and potential risk factors by estimation of unadjusted odds ratios (ORs), univariate logistic regression analyses were performed. For estimation of the association between PHN and individual risk factors while controlling for other risk factors, multivariate logistic regression analysis was used. The potential risk factors included were age group, gender, pre-existing medical conditions, concurrent immunosuppressive therapy, symptoms before rash onset, HZ vaccination status and HZ severity at the first visit.

All statistical analyses were performed using the Statistical Analysis Systems (SAS) version 9.2 on windows.

### 2.5 Ethics

The network ethics committees approved the study protocol, the informed consent forms and other information requiring pre-approval. All enrolled patients in the study gave written informed consent to participate.

## 3 Results

### 3.1 Patient demographics

The study period was from February 2013 to October 2016. Nine networks including 75 GP centers covering an estimated cohort of 43,875 individuals aged ≥50 years were involved for at least part of the study period in the screening of HZ cases for the incidence estimate. Fifty-five GP centers were active in the patient enrolment and follow-up.

A total of 721 individuals aged ≥50 years were screened and diagnosed with a first episode of HZ during the study period, of which 391 were enrolled in the study (according to protocol cohort). Among the screened patients, the proportion of women was 60% and their distribution on pooled age groups was 20%, 29%, 31% and 21% aged 50–59, 60–69, 70–79 and ≥80, respectively.

Among the HZ patients enrolled, the proportion of women was 61% and the cohort distribution on age groups was 22%, 32%, 25% and 21% aged 50–59, 60–69, 70–79 and ≥80, respectively. More than two thirds, 69%, were retired, 17% working, 5% unemployed and 9% recorded their employment status as ‘other’.

The proportion of screened HZ patients enrolled in the study ranged from 45% in the age group 70–79 to 62% in the age group 50–59. The overall proportion enrolled of the screened patients was 55%, the same for women and men.

### 3.2 Overall incidence of HZ in the screened population

The overall incidence was estimated as 6.46 per 1000 PY (95% CI, 5.99–6.95), increasing with age from 3.51 (95% CI: 2.70–4.48) in the group aged 50–54 years to 9.12 (95% CI: 7.50–10.99) in the group aged 75–79 years and 7.11 (95% CI: 6.02–8.34) in those aged ≥80 years (Table 1).

**Table 1:** Estimated incidence of herpes zoster in Italians aged ≥50 years by gender and age.

Age group (years)	Women				Men				Overall			
			Incidence				Incidence				Incidence	
	n	Total person-days	Rate/1000 PY	95% CI	n	Total person-days	Rate/1000 PY	95% CI	n	Total person-days	Rate/1000 PY	95% CI
50–54	49	3,450,586.8	5.19	3.84–6.86	15	3,207,880.5	1.71	0.96–2.82	64	6,664,753.8	3.51	2.70–4.48
55–59	56	3,250,776.8	6.29	4.75–8.17	23	2,882,172.3	2.91	1.85–4.37	79	6,132,949	4.70	3.72–5.86
60–64	39	2,849,623.3	5.00	3.55–6.83	32	2,684,483.5	4.35	2.98–6.15	71	5,534,106.8	4.69	3.66–5.91
65–69	74	2,941,371	9.19	7.22–11.54	62	2,530,581.3	8.95	6.86–11.47	136	5,471,952.3	9.08	7.62–10.74
70–74	54	2,628,036	7.51	5.64–9.79	56	2,191,895	9.33	7.05–12.12	110	4,819,931.0	8.34	6.85–10.05
75–79	71	2,419,504.8	10.72	8.37–13.52	39	1,984,885.3	7.18	5.10–9.81	110	4,404,390.0	9.12	7.50–10.99
≥80	92	4,762,572	7.06	5.69–8.65	59	2,993,205.8	7.20	5.48–9.29	151	7,755,777.8	7.11	6.02–8.34
**≥50**	**435**	**22,302,470.7**	**7.12**	**6.47–7.83**	**286**	**18,475,103.7**	**5.65**	**5.02–6.35**	**721**	**40,783,860.7**	**6.46**	**5.99–6.95**

CI = Confidence interval; n = number of herpes zoster cases; PY = person-years; Total person-days: ∑ (study cohort × study days).

The overall incidence in men and women was 5.65 per 1000 PY (95% CI: 5.02–6.35) and 7.12 per 1000 (95% CI: 6.47–7.83), respectively, with the highest incidence point estimates being 9.33 (95% CI: 7.05–12.12) in men aged 70–74 years and 10.72 (95% CI: 8.37–13.52) in women aged 75–79 years, respectively (Table 1).

### 3.3 Clinical features of the patients with HZ at the initial visit (ATP cohort)

The mean delay between the date of rash onset and the first assessment was 3.0 days (range 0–22 days). At the initial visit 44.7% of the patients indicated to have severe pain (7–10 score) on the ZBPI ‘worst pain’ item 3 (Table 2) and 90% received a prescription for antivirals. Of the 288 patients (73.7% of all), who reported having had symptoms before the onset of the rash, 82.6% reported prodromal pain, 29.2% had experienced malaise and 5.9% fever (Table 2).

**Table 2:** Pain severity and clinical characteristics of the herpes zoster patients at initial visit.

Characteristics	n	%
Severity of HZ pain	N^1^ = 362	
No or mild	43	11.9
Moderate	157	43.4
Severe	162	44.7
Among patients having had symptoms before rash onset	N^2^ = 288	
Prodromal pain	238	82.6
Malaise	84	29.2
Fever	17	5.9
Other symptoms - non-painful	31	10.7
Other symptoms – painful	3	1.0
Patients with co-morbidities at initial visit	N^3^ = 128	
Most frequent pre-existing conditions (>5% of those with such conditions)		
Diabetes mellitus	46	35.9
Current emotional problems, stress or depression	41	32.0
Patients under immunological treatment	*N* = 25	
Oral or parenteral corticosteroids	16	64.0

HZ = Herpes zoster; n = Number of patients concerned; N^1^ = For 29 patients, the initial pain assessment was missing; N^2^ = The number of patients with symptoms before rash onsets; N^3^ = The number of patients with co-morbidities.

Of the 128 patients (32.7% of all) who reported one or more pre-existing medical conditions, 46 (35.9%) had diabetes mellitus and 41 (32.0%) had concurrent emotional problems, stress or depression, both corresponding to the prevalence in the general population of older adults (Epicentro, 2019; EseMed, 2019), and 32 (25.0%) reported other diseases. Twenty-five patients (6.4% of all) were on concurrent immunosuppressive therapy; of these, 16 (64.0%) reported taking oral or parenteral corticosteroids.

HZ-associated complications at the initial visit were reported by 65 patients (16.6%). The most common of these was neurological (mainly persistent HZ-related pain) for 21.0% of these patients, cutaneous (disseminated HZ and bacterial superinfection), 10.7%, and ocular (keratitis), 3.8%.

### 3.4 The proportion of HZ patients developing PHN and their characteristics

The ZBPI questionnaire was completed by 343 (87.7% of the enrolled) patients at Day 90, by 14 (3.6%) at Day 180 and by 4 (1.0%) at Day 270 (Fig. 1). It was completed by 83.7%, 91.0% and 85.8% of patients with initially no or mild pain, moderate pain and severe pain, respectively.

Forty patients (10.23%; 95% CI: 7.41–13.67) developed PHN (Table 3). Of these, 6 (15.0%) reported persistent PHN at Day 180 and 3 (7.5%) still reported ‘worst pain’ ≥3 at Day 270. The proportion of female HZ patients with PHN was 10.97% (95% CI: 7.29–15.66) increasing with age from 7.14% (95% CI: 0.88–23.50) in the age group 50–54 to 14.81% (95% CI: 4.19–33.73) in the age group 75–79. For males, the overall proportion with PHN was 9.09% (95% CI: 5.06–14.78), increasing with age from 9.09% (95% CI: 0.23–41.28) in the age group 50–54 to 16.67% (95% CI: 3.58–41.42) in those aged 75–79 years.

**Table 3:** Proportion of herpes zoster patients with postherpetic neuralgia 90 days after the rash onset by gender and age group.

Age group (years)	Women				Men				Overall			
	N	n	%	95% CI	N	n	%	95% CI	N	n	%	95% CI
50–54	28	2	7.14	0.88–3.50	11	1	9.09	0.23–41.28	39	3	7.69	1.62–20.87
55–59	37	3	8.11	1.70–21.91	11	0	0.00	0.00–28.49	48	3	6.25	1.31–17.20
60–64	18	1	5.56	0.14–27.29	20	0	0.00	0.00–16.84	38	1	2.63	0.07–13.81
65–69	50	7	14.00	5.82–26.74	38	2	5.26	0.64–17.75	88	9	10.23	4.78–18.53
70–74	28	2	7.14	0.88–23.50	25	3	12.00	2.55–31.22	53	5	9.43	3.13–20.66
75–79	27	4	14.81	4.19–33.73	18	3	16.67	3.58–41.42	45	7	15.56	6.49–29.46
≥80	49	7	14.29	5.94–27.24	31	5	16.13	5.45–33.73	80	12	15.00	8.00–24.74
**≥50**	**237**	**26**	**10.97**	**7.29–15.66**	**154**	**14**	**9.09**	**5.06–14.78**	**391**	**40**	**10.23**	**7.41–13.67**

CI = Confidence interval; n = Number of herpes zoster patients of this gender in this age group with postherpetic neuralgia; N = Number of herpes zoster patients of this gender in this age group.

The mean age of the patients with PHN was 73 years, significantly higher than for patients without PHN (69 years, *p* = 0.017). Twenty-six (65.0%) of the patients with PHN were women. Among patients with any pre-existing medical condition, the proportion developing PHN was 11.8%, whereas this proportion was 9.6% among patients with no pre-existing condition.

### 3.5 Predictive factors for PHN

The final logistic regression model showed that the severity of HZ-related pain at the initial visit was a borderline significant predictor for PHN (Table 4). The estimated OR for severe pain versus no/mild pain was OR = 7.28 (95% CI: 0.96–55.57, *p* = 0.05).

**Table 4:** Estimated coefficients of the fitted logistic regression model and the final model for potential risk factors for postherpetic neuralgia.

Model	Variable	Coefficient	Standard error	*P*-Value	OR	95% CI for OR
Saturated model	Age group:					
	60–69 years vs 50–59 years^a^	0.336	0.6474	0.6041	1.399	0.393–4.976
	70–79 years vs 50–59 years^a^	0.777	0.6409	0.2253	2.175	0.619–7.640
	≥80 years vs 50–59 years^a^	0.953	0.6420	0.1378	2.593	0.737–9.125
	Gender Female vs Male^a^	−0.165	0.4055	0.6835	0.848	0.383–1.876
	HZ related complications: Yes vs No^a^	−0.210	0.5319	0.6926	0.810	0.286–2.298
	Current immunosuppressive therapy Yes vs No^a^	0.590	0.7250	0.4155	1.805	0.436–7.474
	Pre-existing medical condition Yes vs No^a^	0.412	0.4063	0.3104	1.510	0.681–3.348
	HZ severity at initial visit					
	Moderate pain vs No or mild pain^a^	0.747	1.0937	0.4947	2.110	0.247–18.000
	Severe pain vs No or mild pain^a^	2.175	1.0512	0.0386	8.801	1.121–69.083
	Timing of treatment with antiviral agent^b^ ≥3 days vs <3 days^a^	0.300	0.3934	0.4454	1.350	0.624–2.919
Final model	HZ severity at initial visit					
	Moderate pain vs No or mild pain^a^	0.608	1.0849	0.5752	1.837	0.219–15.400
	Severe pain vs No or mild pain^a^	1.986	1.0368	0.0555	7.283	0.955–55.567

HZ = herpes zoster; OR = odds ratio; 95% CI for OR = Wald 95% confidence interval; Note: Final model is by considering backward model building strategy with SLSTAY = 0.05 and saturated model is by not considering any model building strategy.

a Reference category.

b Difference between the date of rash onset and the date of first prescription of treatment of antivirals.

## 4 Discussion

This is the first prospective study of the incidence of HZ in Italy focusing on individuals aged ≥50 years. Based on acute HZ cases presenting in the primary care setting in GP practices across the country, an overall incidence in this population of 6.5/1000 PY was estimated. The incidence increased with age up to the age of 79 and then leveled off. The overall incidence was higher for women. Among the HZ patients enrolled in the study, 10% developed PHN with the frequency increasing with age. The only statistically significant risk factor identified for PHN was the severity of pain at the initial visit.

Our estimated overall incidence of HZ in Italian individuals aged ≥50 years is in line with the mean incidence rate of 7–8/1000 PY in this age group reported in a recent systematic review of European studies (Pinchinat et al., 2013). In a recent Italian study, the overall incidence rate was estimated as 6.42/1000 PY (Alicino et al., 2017). Other recent studies performed in Europe with a design similar to ours and focusing on the same population age group show comparable HZ incidence rates and PHN proportions: 6.7/1000 PY and 11.9%, respectively, in Germany (Schmidt-Ott et al., 2018); 6.2/1000 PY and 9.1%, respectively, in immunocompetent individuals, and 7.8/1000 PY and 10.7%, respectively, in immunocompromised individuals in the UK (Yanni et al., 2018).

A previous retrospective study of the same population segment in Italy found an almost identical HZ incidence rate of 6.7/1000 PY but a lower proportion of PHN (at 3 months) of 7.2% (95% CI: 6.2–8.2) (Gialloreti et al., 2010). Gialloreti et al. identified cases of PHN on the basis of prescription of neuropathic pain medication and it is likely to have underestimated the frequency of PHN compared to our prospective collection of patients' experience of pain as recorded in the self-administered ZBPI questionnaires. Using the same method for the identification of PHN cases as Gialloreti, Alicino et al. reported a proportion of PHN at 3 months of 12.7% (Alicino et al., 2017).

Another recent prospective study of Italian HZ patients aged ≥50 made pain assessments during GP visits using a visual analog scale (VAS, 0–10). Defining PHN as the worst pain over the previous 2 weeks ≥ 3 on the VAS at the GP visit at 3 months, they found that 20.6% of the patients had PHN (Bricout et al., 2014). Another publication from this study reported that among the 73% of the enrolled patients who had an underlying medical condition, the proportion with PHN was 20.5%, in contrast to 8.2% for those without (Torcel-Pagnon et al., 2017). The difference between these results and ours underlines the decisive importance of the way PHN is defined and assessed.

About 12% of the study participants did not complete and return the ZBPI at Day 90 and were lost to follow-up. Data presented in the results refers to the enrolled cohort (391 patients), whereas restricting the analysis to those patients who returned the ZBPI questionnaire at Day 90 (343 patients), the proportion with PHN would be 11.7%. On the other hand, it may be hypothesized that patients with severe initial pain, which did not or only weakly respond to treatment, would seek specialist care and drop out of the study for that reason. Whichever way, the completion of the ZBPI questionnaire did not seem to be systematically associated with the intensity of pain at the onset of HZ, with a completion rate at Day 90 for those with no or mild initial pain of 83.7% and 85.8% for those with severe pain. In sum, our approach was conservative and should not lead to an overestimate of the frequency of PHN.

A second limitation of the study is that only a little more than half of the screened patients accepted to be enrolled, a self-selection which may have led to biased estimates. Although the population enrolled was balanced in terms of gender and age, it appears that the severity of acute pain at the initial visit may have been a factor inducing patients to enroll. In fact, the proportion of enrolled among the screened was 44% for those with no or mild pain and 62% for those with severe pain.

Another limitation is that an unknown number of HZ cases may have been missed, in part due to patients not seeking medical care; thus, a frequent hypothetical explanation of the common finding of a higher HZ incidence among women is a gender difference with respect to seeking medical care (Pinchinat et al., 2013). Another part of possibly missed HZ cases may be due to patients seeking specialist dermatological (e.g., hospital emergency) care without seeking primary care attention, and this would lead to an underestimate of the frequency of severe cases. A potential limitation is that the GPs' clinical diagnoses of HZ were not validated by laboratory tests but the large majority of HZ incidence studies, whether prospective or retrospective, rely on clinical diagnosis alone.

Strengths of the study are its prospective cohort design with frequent assessments by the patients using the robust and well-validated ZBPI instrument and establishment of the temporal relationship for PHN and HZ-related complications. A further strength is that the relative contribution of different potential risk factors for developing PHN was assessed by multivariate statistical methods using backward selection procedures.

### 4.1 Study conclusions

**Fig. 2:**
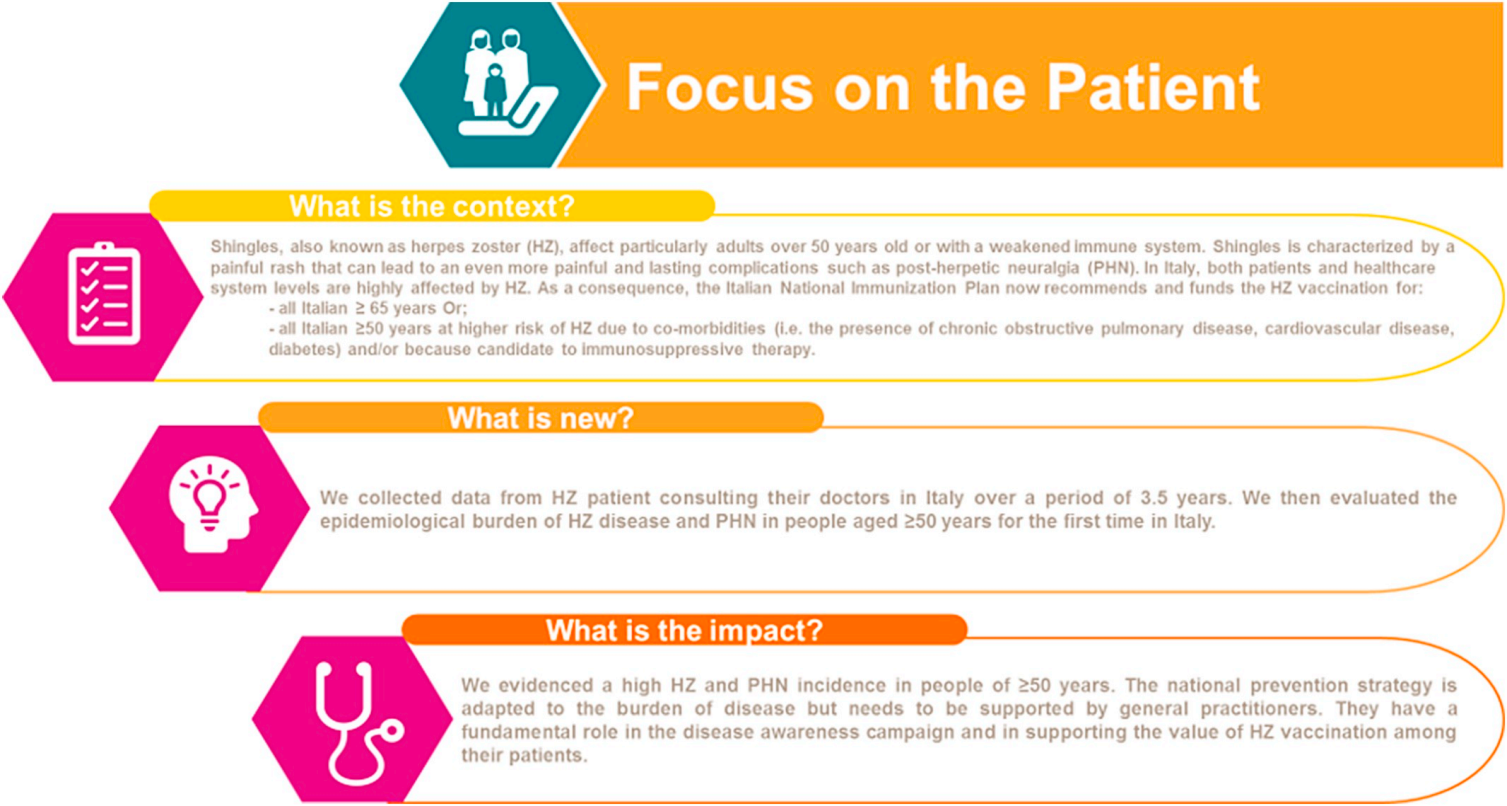
Focus on the patient.

Our results documented the current clinical burden of HZ and PHN in Italian individuals aged 50 years or older. We confirmed the rise of HZ incidence with increasing age and the higher proportion of PHN in the oldest groups. Ongoing demographic changes in Italy, as well as throughout the world, with growing numbers of older adults and also of immunocompromised individuals in all age groups make it likely that the burden of HZ disease will increase considerably in the near future. Appropriate preventive strategies, such as vaccination (cf. the recently adopted national prevention plan in Italy (Ministero della Salute - Italia, 2017)), are available and may reduce the burden of HZ and its associated complications. Fig. 2 presents a summary of the context, outcomes, and impact of this study for healthcare providers.
